# Predicting the risk of future depression among school-attending adolescents in Nigeria using a model developed in Brazil.

**DOI:** 10.1016/j.psychres.2020.113511

**Published:** 2020-12

**Authors:** Rachel Brathwaite, Thiago Botter-Maio Rocha, Christian Kieling, Brandon A. Kohrt, Valeria Mondelli, Abiodun O. Adewuya, Helen L. Fisher

**Affiliations:** a King’s College London, Social, Genetic & Developmental Psychiatry Centre, Institute of Psychiatry, Psychology & Neuroscience, London, United Kingdom; b Department of Psychiatry, Universidade Federal do Rio Grande do Sul, Porto Alegre, Brazil; c Child & Adolescent Psychiatry Division, Hospital de Clínicas de Porto Alegre, Brazil; d Division of Global Mental Health, George Washington University, Washington DC, United States; e King’s College London, Department of Psychological Medicine, Institute of Psychiatry, Psychology & Neuroscience, London, United Kingdom; f National Institute for Health Research Mental Health Biomedical Research Centre, South London and Maudsley NHS Foundation Trust and King's College London, London, United Kingdom; g Department of Behavioural Medicine, Lagos State University College of Medicine, Lagos, Nigeria; h Centre for Mental Health Research and Initiative (CEMHRI), Lagos, Nigeria; i ESRC Centre for Society and Mental Health, King's College London, London, United Kingdom

**Keywords:** Adolescent health, Depressive disorder, Low- and middle-income countries, Mental health, Prediction, Statistical models

## Abstract

Depression commonly emerges in adolescence and is a major public health issue in low- and middle-income countries where 90% of the world's adolescents live. Thus efforts to prevent depression onset are crucial in countries like Nigeria, where two-thirds of the population are aged under 24. Therefore, we tested the ability of a prediction model developed in Brazil to predict future depression in a Nigerian adolescent sample. Data were obtained from school students aged 14–16 years in Lagos, who were assessed in 2016 and 2019 for depression using a self-completed version of the Mini International Neuropsychiatric Interview for Children and Adolescents. Only the 1,928 students free of depression at baseline were included. Penalized logistic regression was used to predict individualized risk of developing depression at follow-up for each adolescent based on the 7 matching baseline sociodemographic predictors from the Brazilian model. Discrimination between adolescents who did and did not develop depression was better than chance (area under the curve = 0.62 (bootstrap-corrected 95% CI: 0.58–0.66). However, the model was not well-calibrated even after adjustment of the intercept, indicating poorer overall performance compared to the original Brazilian cohort. Updating the model with context-specific factors may improve prediction of depression in this setting.

## Highlights

• Pelotas model predicted depression among nigerian adolescents better than chance.
• The model was able to discriminate adolescents with and without depression.
• The model was not well-calibrated indicating poor overall performance.
• Updating the model with context-specific factors may improve depression prediction.

## 1 Introduction

Depression is a serious mental illness and a leading cause of disability globally (World Health Organization [WHO], 2017). Adolescence is the critical developmental period during which symptoms of depression commonly emerge (Avenevoli et al., 2015) and this is particularly problematic in low- and middle-income countries (LMICs) where 90% of the world's adolescents live (Kieling et al., 2011). Moreover, research among adolescents aged 13–17 years across 59 LMICs found ~17% displayed suicidal ideation, planned suicide, or attempted suicide in the previous year (Uddin et al., 2019). Indeed, global estimates ranked suicide as the second leading cause of death among youths aged 15–29 (WHO, 2014). This is compounded by adolescents with depression in LMICs having less access to recommended treatments for depression than adolescents in high-income countries (HIC) (Kakuma et al., 2011) and a lack of understanding of the most appropriate interventions in these different cultural and socioeconomic environments (Kieling et al., 2011). Therefore, it is imperative to understand how to prevent adolescents from developing depression, especially in LMICs where the burden and risk is so high and access to effective treatments is severely limited.

LMICs in the African region are most likely to be affected by the major public health burdens of depression given their large youth populations and limited mental health services. Nigeria is the most populated country in Africa, and an estimated seven million Nigerians had depression in 2015; the highest number of persons with depressive disorders in the African Region (WHO, 2017). Nigeria also had the 15th highest rate of suicides in the world in 2016 (17.3 per 100,000; WHO, 2016). In addition, Nigeria has one of the largest youth populations around the globe, with ~62% of the population younger than 24 years (United Nations World Population Prospects, 2018), who have been confronted with rising youth unemployment rates since 2015 (International Labour Organization ILOSTAT database, 2019). Given the gap between the extent of available services in Nigeria and the large youth population likely to be at risk of depression, it is important to take steps to prevent this disorder and avert its dire consequences in this country.

Predicting the future risk of depression in the adolescent period is one way to enable prevention through facilitating the targeting of interventions at high-risk individuals (Kieling et al., 2019), yet this approach has not been fully explored among adolescents in LMICs (Van Voorhees et al., 2008). Our team have recently developed a model to predict future risk of depression using an adolescent cohort from Brazil, an upper-middle-income country (Rocha et al., 2020). The extent to which this model is able to predict future risk of depression among adolescents in a lower-middle-income country located in a separate continent with different social, economic, and cultural influences will aid understanding of its predictive capacity and potential utility for preventing depression among adolescents in a vastly different environment. In this paper, we therefore aim to test the ability of a prediction model developed in Brazil to predict which adolescents in a sample from Nigeria will go on to develop depression.

## 2 Methods

### 2.1 Description of study setting and recruitment of study cohort

We used longitudinal data from the Lagos Schools Emotional and Behavioural Health Survey (LSEBHS) which collected baseline data during September and November 2016 (Adewuya and Oladipo, 2019) and follow-up data in 2019. This is therefore a secondary analysis of a cross-sectional cohort study. In LSEBHS, one urban and one rural educational district was randomly selected from the two urban and three rural educational districts in Lagos Nigeria at the time. A systematic random sampling method was used to select 47 public senior secondary schools from within the two educational districts, Maryland (urban) and Alimosho (rural). From each of the 47 schools, ~200 students were randomly selected to participate. During the school lunch break, students completed self-administered questionnaires in English (the official language of Nigeria and the main language used in schools), under supervision and assistance from the class teacher or a research assistant. The questionnaires collected information on demographics, family socioeconomic status, violence in the family, substance use, school performance, coping in school, and experiences of physical and mental health problems. In total, 9441 students from 47 senior secondary schools completed the survey in 2016 (4654 males and 4757 females) aged 11–21 years. However, only students who were in year 1 of senior secondary school (SS1) during baseline data collection (*n* = 3499) were followed up in 2019, and in total 3171 students (90.6%) participated at both time-points. Students in the second (SS2) or third (SS3) year of senior secondary school at baseline were not followed up in 2019 because they were expected to have completed school by then and thus be more difficult to recontact.

### 2.2 Ethics and consent

The study was approved by the Ethics and Research Committee of the Lagos State University Teaching Hospital. Written permission was granted by the Lagos Schools Board and the principals of each of the selected schools. Students were given a parent/guardian information sheet which asked the parent/guardian to complete a consent form in English or the preferred local languages of Yoruba, Igbo, and Hausa. A detailed participant information sheet was also given to each student and they completed an assent form to indicate willingness to participate. Only students with both assent and parental consent were eligible to participate (93.4% of those approached).

### 2.3 Measures

#### 2.3.1 Depression assessment

Depression was assessed at baseline and follow-up using an adapted version of the Mini International Neuropsychiatric Interview for Children and Adolescents (MINI-KID; Sheehan et al., 2010). Specifically, the MINI-KID was adapted for self-completion (though the wording of the questions remained unchanged) and evaluated the presence of depressive symptoms in the previous two weeks. Face validity of this adapted version of the MINI-KID was established through review by a local expert panel of child and adolescent psychiatrists, adolescent psychologists, paediatricians, and an expert in psychometrics (see Adewuya and Oladipo, 2019). The specific symptom criteria and clinical cut-off for a DSM-IV diagnosis of major depressive disorder (American Psychiatric Association, 1994) was used to categorize students as depressed at each assessment point (i.e., positive responses to 5 or more items, including a positive response to either depressed mood or anhedonia).

#### 2.3.2 Predictor variables

Within the Nigerian dataset, we *a priori* selected variables that closely matched the 11 predictors used in the Pelotas model. The original predictors were selected to be included in the Pelotas model based on socio-demographic variables that had previously been associated with adolescent depression and that could be easily obtained directly from adolescents and be broadly implemented, thereby improving the probability of successful future implementation in a clinical or educational setting (Rocha et al., 2020). Table S1 in the Supporting Information provides details of how these predictors were defined and assessed in both the Pelotas and Nigerian datasets. Seven predictors were matched in the Nigerian dataset. These were: biological sex, childhood maltreatment, school failure, social isolation, fights, run away from home, and drug use. School failure was determined from the question, ‘Have you ever repeated a class?’ with those answering “yes” considered to have failed school, and “never” as not failing school. Social isolation was assessed from the number of close friends in school, with those with zero close friends considered to be ‘socially isolated’ whereas those with at least 1 close friend were classified as ‘not socially isolated’. Fight involvement was determined from positive answers to both of the following questions: “Have you ever started a fight?” and “Have you ever hurt someone on purpose with or without a weapon?”. A “yes” response to ‘Have you ever run away from home for many days?’ was used to determine that the adolescent had run away from home. Responses to questions on how frequently do you take any of the following: alcohol (beer/stout, ogogoro, schnapps, paraga, alomo bitters), cigarettes (Rothmans, White London etc.) and Marijuana/ Igbo/ Indian Hemp/ Weed/ Hemp were used to determine drug use. Participants were categorized into those who did not use vs those who used a little or a lot. Childhood maltreatment of differing degrees was determined from responses to questions on lifetime exposure to domestic violence and physical abuse, and whether they had lived with a primary biological caregiver (if not then this was used to approximate potential neglect). Participants who lived with at least one parent and were not exposed to domestic violence or physical abuse were considered to have not experienced childhood maltreatment, while childhood maltreatment was considered probably likely if participants answered “yes a little” to experiencing both domestic violence and physical abuse or not living with any biological parent. Severe childhood maltreatment was considered present if participants answered “yes a lot” to any question on domestic violence or physical abuse.

### 2.4 Statistical analysis

For this analysis, from the 3171 participants who completed both the baseline and follow-up assessments, we selected those who were aged 14 to 16 years at baseline (*n* = 2321), in order to be as close as possible to the age-15 baseline assessment conducted in the original Pelotas sample while still retaining a reasonable sample size. We then excluded those participants who met criteria for depression at baseline (*n* = 393) as this was an exclusion criterion in the Pelotas sample. Therefore, this resulted in a total of 1928 students (976 males and 952 females) being included in the final analysis (see Figure S1 for a flowchart explaining the selection of the final sample).

STATA was used for data management (StataCorp, 2017), and analyses were conducted in R (R Core Team, 2019). A logistic regression predictive model developed using penalized maximum likelihood estimation (to minimize overfitting) was developed in the 1993 Brazilian cohort comprising 11 predictors of adolescent depression (Rocha et al., 2020). This model was rebuilt removing the four predictors that were not available in the Nigerian dataset. We then used this model to predict depression in the Nigerian sample (standard external validation). The model's intercept was then adjusted to account for the mis-calibration which commonly occurs when a model is validated in a dataset different from the one it was developed in (adjusted external validation). The regression coefficients of the model's predictors were then re-estimated in the Nigerian sample (refitted model).

The performance of the model in the Nigerian dataset was evaluated via its discrimination and calibration. Discrimination, or the model's ability to separate high risk and low risk individuals, was assessed by the area under the receiver operating characteristic curve (AUC) (Steyerberg et al., 2010). An AUC value of 0.50 represents a model that discriminates between adolescents with and without depression at follow-up no better than chance, while an AUC of 1 represents a model that discriminates between those with and without depression perfectly (Hosmer et al., 2013). We evaluated the agreement between model predictions and the actual observed rates of depression in the Nigerian sample visually using calibration plots and from the calibration-in-the-large and calibration slope statistics (Fenlon et al., 2018). The calibration-in-the-large (or intercept) is a measure which compares the average of all the predicted probabilities in the model with all observed cases of depression. The closer the calibration-in-the-large is to zero the better the model. The calibration slope measures the degree of agreement between model predictions and observed rates of depression. A perfect model has a calibration slope of 1, and intercept of 0, depicted by a 45° line on the calibration plot. A chi-square test to measure unreliability of the calibration accuracy was performed to assess whether there was a statistically significant difference between the model predictions and the ideal 45° line (Fenlon et al., 2018). The value of the Brier score was used to give an indication of the overall performance of the model (Steyerberg et al., 2010). The Brier score represents the average squared difference between the model predictions and the actual rates of depression observed in the sample (Brier, 1950). The closer the Brier score is to zero, the better the model.

Note that penalized regression models are different from traditional regression models since a penalty is applied to the size of the regression coefficients causing estimates of the predictor coefficients to be biased in order to improve overall model predictions. Therefore, we will not focus on assessing predictor coefficients in the model separately, but rather on how the model performs as a whole with the combination of predictors.

Because the sample had a wide age range at baseline, we ascertained if different assessment ages at baseline affected the prediction of the model. To make the assessment age comparable to what was done when analysing the Pelotas sample, we restricted the Nigerian sample to adolescents only aged 15 at baseline for a sensitivity analysis. We also decided to utilize the entire age range by including all adolescents aged 17 or younger at baseline in subsequent sensitivity analyses.

## 3 Results

### 3.1 Description of sample population

A total of 1928 students aged 14 to 16 years at baseline were included in the final analysis, approximately half of whom were male (50.6%). These students attended 26 senior secondary schools within the Alimosho and Maryland educational districts in Lagos and were in the last 3 years prior to completing the Ordinary Level (school-leaving) examinations. Approximately 55.8% attended schools located in rural areas, 10.9% in urban areas, and 33.4% in semi-urban areas. Students normally attend schools in close proximity to their place of residence. The prevalence of depression at follow-up in this sample was 11.8% (95% CI 10.4% to 13.3%). In terms of predictors, school failure was present among approximately one quarter of the student sample (25.8%), whereas getting into physical fights (3.9%), drug use (6.0%), social isolation (4.4%), and running away from home (2.3%) were less common. Few students were categorized as having experienced severe childhood maltreatment (6.7%), while 18.4% were considered to have probably experienced childhood maltreatment.

### 3.2 External validation of the Brazilian model in the Nigerian cohort

**Fig. 1:**
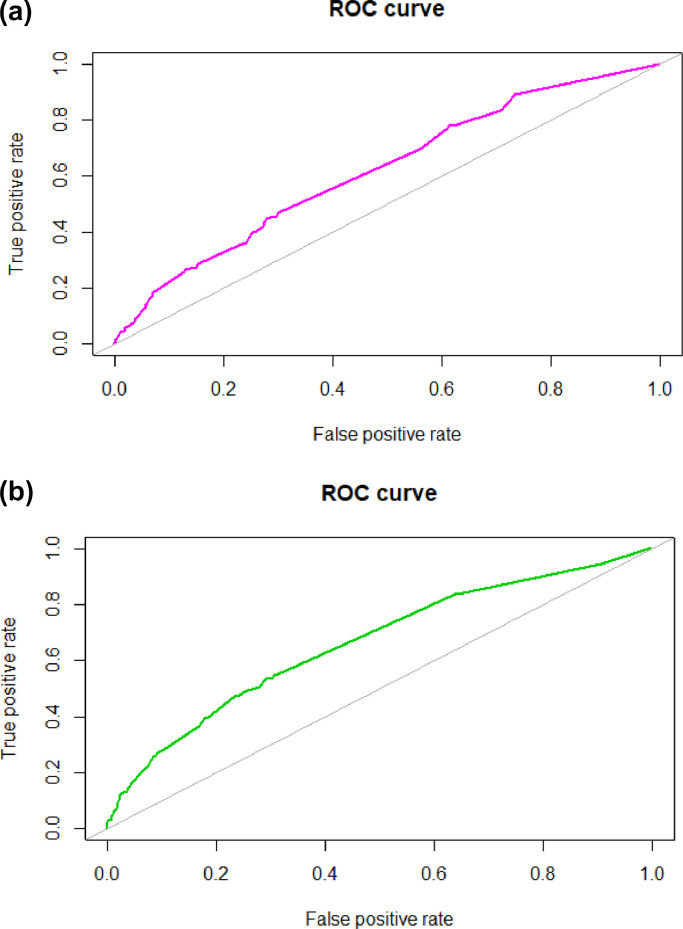
**ROC curve for a) the Pelotas model externally validated in the Nigerian data (area under the curve (AUC) =0.62; bootstrap-corrected 95% confidence interval [CI]: 0.58 – 0.66), and b) the Pelotas model refitted in the Nigerian data (AUC=0.66; bootstrap-corrected 95% CI: 0.63 – 0.70)**. The y-axis shows the true positive rate: the proportion of adolescents correctly identified with depression at follow-up. The x-axis shows the false positive rate: the proportion of adolescents who were wrongly identified as having depression at follow-up. The gray diagonal line represents a model that discriminates the same as chance.

The Pelotas model's discriminative capacity in the Nigerian sample was lower than in the Brazilian sample where the model was developed (Table 1). However, the Pelotas model's capacity to discriminate between Nigerian students with and without depression was better than chance (AUC=0.62; bootstrap-corrected 95% CI: 0.58, 0.66) (Fig. 1a). Its overall performance was worse than its performance in the Pelotas dataset, as indicated by the higher Brier score (Table 1). When the model was refitted in the Nigerian dataset, its discrimination improved slightly with an AUC of 0.66 (bootstrap-corrected 95% CI: 0.63 – 0.70; Fig. 1b, Table 1).

**Table 1:** Comparison of performance metrics for the Pelotas model when externally validated in the Nigerian cohort compared to its apparent and internal validation in the Pelotas cohort.

		Pelotas cohort		Nigerian cohort		
Model assessment	Performance measures	Apparent validation	Internal validation	Standard External validation	Adjusted External validation	Refitted model
**Overall performance or model fit**	**Brier score**	0.03	0.03	0.11	0.10	0.10
**Discrimination**	**AUC (95% CI)**	0.78 (0.73 - 0.82)	0.71 (*)	0.62 (0.58, 0.66)	0.62 (0.58, 0.66)	0.66 (0.63, 0.70)
**Calibration**	**Calibration-in-the-large**	0.00	0.02	1.96	0.00	0.00
	**Calibration slope**	1.26	1.00	0.64	0.64	1.07

Note: 86.3% of original Pelotas model information available for external validation in the Nigerian cohort due to the availability of only 7 of the 11 predictors in the Nigerian dataset.

Apparent validation: the performance of the Pelotas model comprising 11 predictors in the Pelotas cohort.

Internal validation: evaluation of the performance of the Pelotas model comprising 11 predictors after controlling for overfitting, using bootstrapping techniques. Bootstrapping is a procedure in which random samples of the same size as the original dataset are repeatedly drawn from the original dataset. This creates a wide range of possible samples that could have been drawn for the study with various combinations of values providing an estimate of the variance existing between random samples drawn from the same population. Internal validation represents the likely performance of the model in new subjects.

Standard external validation: evaluation of the performance of the Pelotas model comprising 7 predictors when applied to the Nigerian sample.

Adjusted external validation: Performance after the intercept was corrected and applied to the Nigerian sample.

Refitted model: regression coefficients of the Pelotas model comprising 7 predictors re-estimated in the Nigerian dataset.

AUC: area under the curve of the receiver operating characteristic (presented as a proportion). The AUC is identical to the C-statistic for binary outcomes.

Brier score: a goodness-of-fit test used for binary or categorical values. It is calculated by squaring the difference between each binary outcome and its predicted probability and taking the average - a Brier score of 0 represents a perfect model.

Calibration-in-the-large (model intercept) compares the average of all the predicted probabilities in the model with all observed cases of depression. The closer the calibration-in-the-large is to zero the better the model.

Calibration slope of a perfect model is equal to 1. A calibration slope <1 indicates overfitting of the coefficients in the model and >1 indicates underfitting.

⁎ Unable to derive 95% confidence interval for the internal validation.

**Fig. 2:**
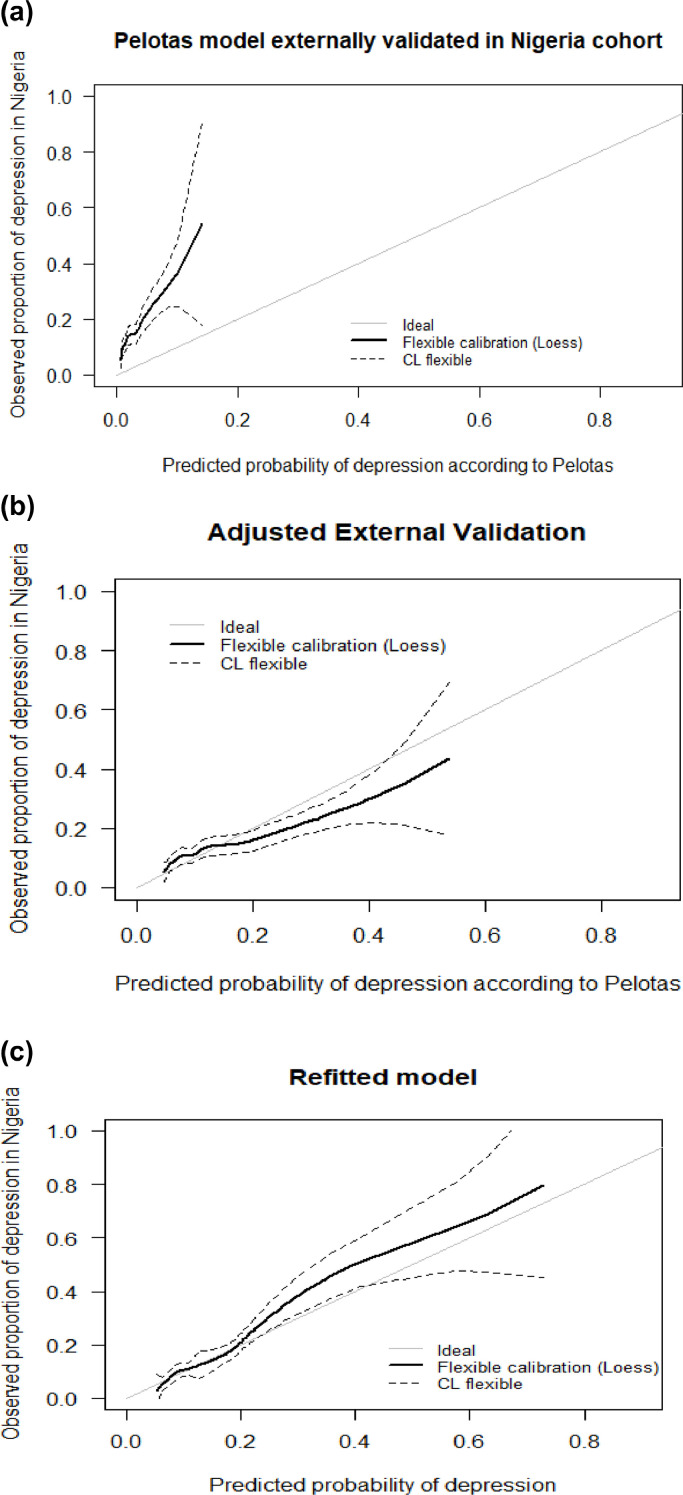
**Calibration plot for: a) the Pelotas model externally validated in the Nigerian dataset, b) when the intercept of the Pelotas model was adjusted and then applied to the Nigerian dataset, and c) the refitted model (model coefficients re-estimated in the Nigerian sample aged 14–16)**. Graphical display of model predictions on the x axis (as depicted by the black flexible calibration line with 95% confidence limits around the model predictions as indicated by the dashed lines) and observed proportion of depression in the Nigerian sample on the y axis. The calibration plots show how well the model predictions align with the observed rate of depression in the Nigerian sample at follow-up. Perfect agreement between the predictions and the observed rate are indicated by perfect alignment on the light gray ideal line.

The agreement between the model's predictions and the observed frequency of depression in the Nigerian sample was initially poor (Fig. 2a), but its calibration improved when the intercept was adjusted (Fig. 2b). The model's overall goodness-of-fit also improved after adjustment of the intercept as indicated by the lower Brier score (Table 1). However, there still appeared to be some degree of mis-calibration (Fig. 2b). At probabilities between 0.2 and 0.4, the model predictions were higher than the actual observed proportion of depression in the Nigerian sample. Wider confidence intervals were also observed at values larger than 0.4 possibly due to only a few adolescents with very high predicted probabilities of depression in the sample. The calibration slope was 0.7 and since this is less than 1 it indicates some degree of overfitting by the model coefficients (Harrell, 2015). The p-value from the chi-square test with 2 degrees of freedom, which tests for unreliability of the calibration accuracy, was significant (χ^2^=12.52, *p* = 0.002). This confirms that there was a statistically significant difference between the model predictions and the ideal 45° line, indicating that there was evidence of imperfect calibration. The refitted model's calibration was similar to the Adjusted Pelotas model, except it underestimated rather than overestimated risk of depression between probabilities of 0.2 and 0.4 (Fig. 2c). Its overall performance was similar to the Adjusted model, with a Brier score of 0.10 (Table 1).

### 3.3 Sensitivity analysis findings

There was not much difference in the model's discrimination when the analysis was restricted to students aged 15 at baseline (*N* = 768; AUC=0.62; bootstrap-corrected 95% CI: 0.56 - 0.67) as well as when students aged 17 years or younger were included (*N* = 2511; AUC=0.61; bootstrap-corrected 95% CI 0.58 - 0.65) (see Supporting Information Table S2, and Figures S2 and S3). In the sample of adolescents aged 15 years only, the adjusted model predictions seemed better aligned to the actual observed rate of depression in the sample between values 0.05 to 0.30 (Figure S4b) than the unadjusted model (Figure S4a). However, in the sample aged ≤17 years, the adjusted model's calibration was comparable to the original sample aged 14–16 years (Table S2), since it appeared not to be well-calibrated for probabilities within the range 0.2 to 0.4 (Figure S5b), although it was still better calibrated than the unadjusted model (Figure S5a). For both populations, the refitted models’ predictions were in good agreement with the observed rates of depression, and confidence intervals included the ideal line across the range of predicted probabilities (Figures S4c and S5c).

## 4 Discussion

The prediction model developed in the 1993 Pelotas cohort, reduced from eleven to seven predictors after data harmonization (biological sex, drug use, ran away from home, school failure, fights, childhood maltreatment, and social isolation), was able to predict future depression in an adolescent student sample in another continent in Lagos, Nigeria, better than chance. There was a 62% (58.1% to 65.7%) chance that a randomly selected Nigerian adolescent who developed depression would have a higher risk score than a randomly selected Nigerian adolescent who did not develop depression. However, we were unable to achieve good calibration (Van Calster et al., 2019) even after adjustment of the model's intercept (Steyerberg et al., 2004). The model predictions overestimated the true observed rate of depression in the Nigerian sample at probability values between 0.2 and 0.4. In this case, extending the model to incorporate additional predictors may be more beneficial in achieving better calibration and more accurate predictions (Steyerberg et al., 2004). Cautious interpretation of the current model predictions is thus recommended.

The discriminative ability of the Pelotas model externally validated in the Nigerian sample was not as good as the Framingham risk score used to predict cardiovascular disease (AUC: for men=0.76; and women=0.79) (D'Agostino et al., 2008). The AUC was also lower than when the Pelotas model was validated in another LMIC adolescent cohort in Nepal (AUC=0.72; bootstrap corrected 95% CI 0.62 – 0.83) (Brathwaite et al., 2020). However, despite the loss in discrimination and calibration, the findings support its ability to predict depression reasonably well in adolescent populations in different geographical locations with different cultural and socioeconomic backgrounds.

The lower performance achieved could be due to overfitting. An overfitted model is one that fits the development data too closely (in this instance too much random noise could have been captured in the Pelotas sample), thus when applied to an external dataset the model shows lower discrimination, overestimating those at high risk of depression and underestimating those at low risk of depression (Van Calster et al., 2019). However, the use of the penalized logistic regression method for the Pelotas model development (Rocha et al., 2020), reduces overfitting and increases the likelihood of achieving reliable results in external validation (Harrell, 2015). It is more plausible that differences in how depression was measured, the inclusion criteria for study recruitment and the final analysis sample, and the availability of predictors and how they were defined may explain the lower performance. For example, the prevalence of depression in the Pelotas sample was 3.1% while in the Nigerian sample it was 11.8%. The higher prevalence of depression in the Nigerian sample might have been due to the use of the MINI-KID as a self-report instrument rather than a clinical interview (which was conducted in the Pelotas sample) thus reducing the comparability of the samples and potentially leading to an over-estimation of depression in the Nigerian sample and limiting generalizability. Only face validity of the MINI-KID adapted for self-completion was established (see Adewuya and Oladipo, 2019) and so other psychometric properties of this adapted version remain unknown. Moreover, a lower event rate in the development sample can lead to overestimation when externally validated in a new sample with higher event rates (Collins and Altman, 2012; Van Calster et al., 2019).

Another difference is that the Nigerian adolescents in this study were recruited from schools, while those in Brazil were assessed even if they were not attending school. A quarter of adolescents in Nigeria are either out of formal education or with low attendance rates (UNICEF Nigeria, 2017) and may be at higher risk of developing depression or have different risk factors than those included in this study. Therefore, our findings cannot be generalized to adolescents in Nigeria more broadly. Additionally, participants with either intelligent quotient <70 and no pubertal signs by age 15 were excluded from the final Pelotas sample for analysis, but this data was not collected in the LSEBHS, preventing exclusion of such participants which may have limited the model's performance. Furthermore, there were differences in the age of adolescents at baseline between the two samples, although restricting the Nigerian baseline sample to different age ranges did not hugely influence the model's discrimination and calibration.

The missing predictor variables (skin color, relationship with mother, relationship with father, and relationship between parents) in the Nigerian dataset are likely important explanatory variables which may have improved the calibration of the model, and thus their omission is a limitation of this study. Indeed, poor family functioning has previously been shown to be a strong predictor of adolescent depression within a Nigerian sample (Adewuya and Ologun, 2006). Additionally, cultural and environmental differences between Brazil and Nigeria (e.g., differences in the mental health impact of urban and rural areas in terms of social inequality, youth unemployment, housing, sanitation, and air quality; Leithead, 2017) may play a role in how well the model predicts depression in the Lagos sample. One way to address the different influences of the social, economic, and cultural factors between the development sample environment and the external validation environment is to incorporate information specific to the Nigerian environment into the model to see how it improves its performance. This can be done using net reclassification improvement methods which assess to what extent individuals are correctly reclassified into risk categories following the inclusion of new variables into the model (Pencina et al., 2017). For example, to update the model in the Nigerian setting, family functioning could be added as a new predictor in the model. If improvement in model performance is observed, then one can say that the model containing information specific to the new environment was better than the original model at predicting risk for adolescent depression.

Preventing depression among adolescents in a country with vastly diverse populations and economic challenges is inevitably difficult. We recognize that the adolescents who participated in the LSEBHS do not represent the socio-cultural variability within Nigeria and therefore the use of a single model to predict depression for all Nigerian adolescents would not necessarily be appropriate. However, given the lack of mental healthcare resources, innovative use of non-specialist mental health-trained staff in non-medical environments should be considered to provide targeted interventions for adolescents at elevated risk of depression. If the depression risk screening tool was to be implemented in Nigerian secondary schools, for example, it would be essential to first ensure that appropriate structures, support systems, and trained staff are present within the school environment so that adolescents can receive appropriate interventions, care, and timely referrals after they are identified as being at high risk of depression. Exploration of how feasible and acceptable this initiative is with key stakeholders in Nigeria is also required before an intervention such as this depression risk screening tool is implemented. Perhaps also incorporating mental health education into Nigerian secondary schools could help sensitize young people to the mental health challenges faced by many adolescents and promote a supportive environment.

To facilitate detection, prevention, and treatment of adolescent mental illness, the Lagos State government recently actioned the development of the Lagos State Mental Health Policy, which includes several key objectives including a focus on: fighting stigma and discrimination towards people suffering with mental health problems; improving awareness; and encouraging early detection of mental health problems in children and adolescents (Lagos State Government Ministry of Health, 2017). To complement these efforts, the risk prediction model developed in this paper could assist with facilitating early detection of depression among Nigerian adolescents. The next steps to make risk prediction possible in Nigeria are further model validation, model improvements, and assessment of its feasibility and likely acceptability.

In conclusion, we were able to reasonably predict future risk of depression in an adolescent student sample in Lagos, Nigeria, using a predictive model developed in Brazil. This adds support to the transcultural applicability of the model to be used as a tool for early identification of depression risk among adolescents. However, further fine tuning of the model to improve its calibration in other Nigerian settings is required before it could be used in practice to identify adolescents at risk of depression in this context.

## Funding

The IDEA project is funded by an MQ Brighter Futures grant [MQBF/1 IDEA]. Additional support was provided by the UK Medical Research Council [MC_PC_MR/R019460/1] and the Academy of Medical Sciences [GCRFNG\100281] under the Global Challenges Research Fund. The Lagos Schools Emotional and Behavioural Health Survey (LSEBHS) was funded by the Lagos State Ministry of Health. The Centre for Mental Health Research and Initiative (CEMHRI) executed the LSEBHS in schools. Dr Fisher is supported by a British Academy Mid-Career Fellowship [MD\170005] and by the Economic and Social Research Council (ESRC) Centre for Society and Mental Health at King's College London [ES/S012567/1]. Dr Kieling is a Conselho Nacional de Desenvolvimento Científico e Tecnológico (CNPq) researcher and an Academy of Medical Sciences Newton Advanced Fellow. Dr Mondelli is supported by the Medical Research Foundation [MRF-160–0005-ELP-MONDE] and by the National Institute for Health Research (NIHR) Biomedical Research Centre at South London and Maudsley NHS Foundation Trust and King's College London. The views expressed are those of the authors and not necessarily those of the NHS, the NIHR, the Department of Health and Social Care, the ESRC, or King's College London.

## ROLE of the funding SOURCE

These funders played no role in study design; in the collection, analysis and interpretation of data; in the writing of the report; nor in the decision to submit this article for publication.

## CRediT authorship contribution statement

**Rachel Brathwaite:** Conceptualization, Data curation, Formal analysis, Writing - original draft. **Thiago Botter-Maio Rocha:** Conceptualization, Methodology, Writing - review & editing. **Christian Kieling:** Conceptualization, Funding acquisition, Methodology, Writing - review & editing. **Brandon A. Kohrt:** Conceptualization, Writing - review & editing. **Valeria Mondelli:** Conceptualization, Funding acquisition, Writing - review & editing. **Abiodun O. Adewuya:** Conceptualization, Funding acquisition, Investigation, Writing - review & editing. **Helen L. Fisher:** Conceptualization, Funding acquisition, Supervision, Writing - review & editing.

## Declaration of competing interest

Dr Mondelli has received research funding from Johnson & Johnson, a pharmaceutical company interested in the development of anti-inflammatory strategies for depression, but the research described in this paper is unrelated to this funding. All other authors declare they have no conflicts of interest to report.

## Appendix Supplementary materials

Image, application 1

Image, application 2
