# Supplementary material for: Predicting the risk of future depression among school-attending adolescents in Nigeria using a model developed in Brazil
Source: Psychiatry Res. 2020 Dec;294:113511. doi: 10.1016/j.psychres.2020.113511 (PMC7732701; doi:10.1016/j.psychres.2020.113511)
Supplement: Supplementary file 1 [file mmc1.docx]

**AUTHORS’ CONTRIBUTIONS**

**Rachel Brathwaite:** Conceptualization; Data curation; Formal analysis; Writing - original draft. **Thiago Botter-Maio Rocha**: Conceptualization; Methodology; Writing - review & editing. **Christian Kieling:** Conceptualization; Funding acquisition; Methodology; Writing - review & editing. **Brandon A. Kohrt:** Conceptualization; Writing - review & editing. **Valeria Mondelli:** Conceptualization; Funding acquisition; Writing - review & editing. **Abiodun O. Adewuya:** Conceptualization; Funding acquisition; Investigation; Writing - review & editing. **Helen L. Fisher:** Conceptualization; Funding acquisition; Supervision; Writing - review & editing.
